# Exploring neurodegenerative disorders using a novel integrated model of cerebral transport: Initial results

**DOI:** 10.1177/0954411920964630

**Published:** 2020-10-20

**Authors:** John C Vardakis, Dean Chou, Liwei Guo, Yiannis Ventikos

**Affiliations:** 1 CISTIB Centre for Computational Imaging and Simulation Technologies in Biomedicine, School of Computing, University of Leeds, Leeds, UK; 2 Department of Biomedical Engineering, National Cheng Kung University, Tainan City, Taiwan; 3 Department of Mechanical Engineering, University College London, London, UK

**Keywords:** Glymphatic system, multiple-network poroelastic theory, neurovascular unit, finite element method, Alzheimer’s disease

## Abstract

The *neurovascular unit* (NVU) underlines the complex and symbiotic relationship between brain cells and the cerebral vasculature, and dictates the need to consider both neurodegenerative and cerebrovascular diseases under the same mechanistic umbrella. Importantly, unlike peripheral organs, the brain was thought not to contain a dedicated lymphatics system. The *glymphatic system* concept (a portmanteau of glia and lymphatic) has further emphasized the importance of cerebrospinal fluid transport and emphasized its role as a mechanism for waste removal from the central nervous system. In this work, we outline a novel multiporoelastic solver which is embedded within a high precision, subject specific workflow that allows for the co-existence of a multitude of interconnected compartments with varying properties (multiple-network poroelastic theory, or MPET), that allow for the physiologically accurate representation of perfused brain tissue. This novel numerical template is based on a six-compartment MPET system (6-MPET) and is implemented through an in-house finite element code. The latter utilises the specificity of a high throughput imaging pipeline (which has been extended to incorporate the regional variation of mechanical properties) and blood flow variability model developed as part of the VPH-DARE@IT research platform. To exemplify the capability of this large-scale consolidated pipeline, a cognitively healthy subject is used to acquire novel, biomechanistically inspired biomarkers relating to primary and derivative variables of the 6-MPET system. These biomarkers are shown to capture the sophisticated nature of the NVU and the glymphatic system, paving the way for a potential route in deconvoluting the complexity associated with the likely interdependence of neurodegenerative and cerebrovascular diseases. The present study is the first, to the best of our knowledge, that casts and implements the 6-MPET equations in a 3D anatomically accurate brain geometry.

## Introduction

Although the brain only accounts for approximately 2% of the body’s total mass, it has the capacity to consume approximately one fifth of its resting energy production.^1^ The high metabolic demand of this organ is driven by a constant flow of blood supplied through a widespread and densely packed network of microvasculature (the sensitivity of the vascular tone is essential in regulating the cerebral blood supply) comprising of arterioles, capillaries and venules. The architectonics of the microvasculature in the cerebral cortex allows for the adequate supply of oxygen and nutrients^2^ and is also unique in its ability to form a blood–brain barrier (BBB). This barrier regulates homeostasis of the central nervous system (CNS) by tightly regulating the transport of ions, molecules and cells. The BBB does not function independently, as it is a constituent of a multi-cellular neurovascular unit (NVU). The NVU comprises of neurons, astrocytes, pericytes, microglia and blood vessels. In this work, a highly integrated workflow that can capture the broad spectrum of biological flows within complex three-dimensional, deformable and permeable brain tissue is presented. The core numerical template that sits at the epicentre of this state-of-the-art precision medicine pipeline was largely developed within the VPH-Dementia Research Enabled by IT Project.^3^ In this project, the focus was to extend the understanding of Alzheimer’s Disease (AD) in addition to promoting earlier differential diagnosis through a unified multiscale modelling platform that accounts for a subjects’ environment and lifestyle. In the work presented here, the original four-compartment poroelastic model presented as part of this precision medicine pipeline is extended to six-compartments. This extension is important, since the four-compartment model made an implied assumption regarding cerebrospinal fluid (CSF): it amalgamated all fluid outside the vascular tree into a single entity, or one compartment, within the multiporoelastic model. A physiologically more accurate representation requires the intracellular and extracellular fluid to be separated, in addition to incorporating distinctive extracellular fluid pathways, like the glymphatic system,^4^ which require special treatment in order to more faithfully represent both the complex hypotheses and the pathophysiological processes allied to neurodegenerative diseases such as AD.

### The neurovascular unit: Origins and composition

**Figure 1.**
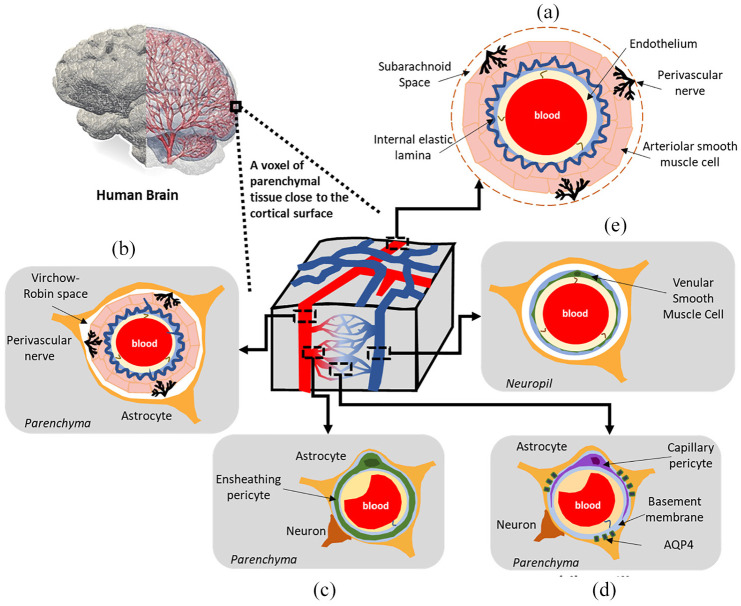
A schematic representation of the mature (adult) neurovascular unit (NVU) within a rodent cerebral cortex. A range of vessels are situated within parenchymal tissue. The vascular wall composition at the level of (a) the pial arterioles on the cortical surface, (b) the penetrating arterioles, (c) pre-capillary arterioles, (d) capillaries, and (e) the ascending venules. AQP4: aquaporin 4.

The conceptual understanding of the neurovascular unit (NVU) underlines the complex and symbiotic association between brain cells and the cerebral vasculature. More specifically, the NVU dictated the need to consider both neurodegenerative and cerebrovascular diseases under the same mechanistic umbrella, by offering the opportunity for important discussions revolving around the brain and its vessels in both healthy and diseased states.^5^ Figure 1(a)–(e) depicts the range of vessels that are situated within parenchymal tissue. For a thorough treatment of the architecture of these vessels along with the important roles these play in fluid transport in the brain, the reader is referred to the work of Iadecola, McConnell et al. and Coelho-Santos and Shih.^5–7^

### Understanding dementia and Alzheimer’s Disease

By 2050, it is estimated that over 150 million people will be living with dementia.^8^ Alzheimer’s Disease (AD) is acknowledged as a progressive multifarious neurodegenerative disorder. It is the most prevalent form of dementia (accounting for around 50%–70% of cases) and is the main cortical neurodegenerative disease.^9^ With an ageing global population, AD is projected to generate a major a public health crisis in the coming decades.^10^ A more detailed understanding of the mechanisms driving AD necessitates the redefinition of the disorder as a mixture of dysfunctions rather than one defining pathology.

### The vascular hypothesis of AD and the role of hypoperfusion

From a pathophysiological perspective, several hypotheses allied to AD have been studied to help understand this multifactorial disorder (amyloid cascade, cholinergic and the vascular hypotheses).^11,12^ The well documented pathological hallmarks of AD incorporate amyloid-β peptide (Aβ) plaques, tau tangles, neuroinflammation, and subsequent neurodegeneration.^10^ Aβ can be found both in the brain parenchyma, or in and around blood vessels (cerebral amyloid angiopathy).^13^ A competing hypothesis (to the amyloid cascade hypothesis) is the vascular hypothesis of AD. This hypothesis is reinforced by increasing evidence that relates vascular dysfunction and AD development.^9,14^ The latter is very often associated with cerebrovascular pathologies^12,14–18^ (such as microinfarcts, haemorrhage, decreased cerebral blood flow, small vessel disease and white matter abnormalities).^19,20^ It has been observed in recent studies that there is a correlation between cognitive dysfunction and vascular disease.^18,21^ Additionally, numerous investigations fortify the association between various vascular risk factors (such as arterial hypertension,^22^ obesity,^23^ atherosclerosis^24^ and genetic risk factors (for example the APOE ε4 allele))^25^ and the heightened probability of developing AD. Recent studies postulate that cerebral hypoperfusion (CH) precedes AD.^26,27^ CH triggers a metabolic energy crisis (via the promotion of a hypoxic state and the accumulation of Aβ and aggravation of vascular disease) which ultimately leads to neuronal degradation.^9,28–33^

### Clearance pathways for unwanted molecules in the brain: The glymphatic system concept

Research into cerebral health and disease has attracted increasing attention, yet, the exact clearance mechanisms responsible are yet to be identified. Unlike peripheral organs, the brain was thought not to contain a dedicated lymphatics system.

The glymphatic system concept has given new impetus to the field of CSF fluid transport and underscored its role as a process for waste clearance from the CNS.^34^ This hypothesis involves CSF being continuously transported from the basal cisterns and into the subarachnoid space (SAS) at the periphery of the cerebral hemispheres, to the periarterial spaces. Subsequently, CSF is driven from the periarterial network into the interstitial fluid (ISF) space. This transport of fluid is seemingly facilitated via aquaporin-4 (AQP4) water channels. The latter are predominantly localised on astroglia end-feet (see Figure 1(d)).^35^ The latter process enables the mixing of both CSF and ISF in addition to waste solute removal. Lastly, the CSF and ISF mixes with interstitial waste solutes, and is transported towards the perivenous compartment of the larger central veins, ultimately entering the systemic circulation. ^34,36^

Recent studies on the downstream lymphatic network, and how this interconnect with the glymphatic pathway have become important areas of consideration.^37–39^ As described by Benveniste et al., the glymphatic system may be viewed as a waste processing unit,^34,40^ with the key constituents being: perivascular AQP4 water channels, hydraulic forces associated with arterial pulsation, respiratory effort, and body position,^34,41–43^ regular CSF production and transport, and a state of arousal.^44,45^

The convoluted nature of the glymphatic system along with the visibly complex, and broad spectrum of disorders that constitute dementia requires the use of a biomechanistic model that is both accurate and general enough to define clinically relevant biomarkers allied to the multiscale cerebral environment. A precision medicine workflow is presented here, which allows for a previously unexplored understanding of the disease trajectory (from mild cognitive impairment [MCI] to AD) through the lens of the physiologically accurate definition and interplay of both the NVU and the glymphatic pathway (by treating this pathway as a separate compartment within a multiporoelastic system). The *in-silico* model presented here requires the use of a surrogate model that outputs arterial blood flow waveforms (that are used as boundary conditions) whilst taking into account lifestyle and environmental factors (LEFs) and subject profiles. Additionally, an imaging pipeline is used to further personalize the workflow via the acquisition of: (i) a subject-specific atlas of CSF/extracellular fluid (ECF) permeability within the cerebral parenchymal tissue; (ii), the accurate representations of the cerebroventricular geometries; and (iii), the partitioning of white and grey matter which is complemented by a field of varying parenchymal tissue stiffness. The reader is referred to^3,46–48^ for further details.

### Outline of the article

The methodology behind the full implementation of the novel six compartment poroelastic model follows in the ‘Methodology’ section, which also briefly describes the extension (spatial variation of the region-specific mechanical properties of brain tissue) of a recently developed high precision workflow.^3^ The results, discussion and limitations are given in the ‘Results and discussion’ section, where 6-MPET results at the level of the neurovascular unit for five separate regions of the brain (cortical grey matter, hippocampus, amygdala, thalamus and brainstem) are presented. The conclusions and perspectives for future work are given in the ‘Conclusion’ section.

## Methodology

### The six-compartment multiporoelastic model for perfused parenchymal tissue

The underlying conservation equations allied to multiple network poroelastic theory (MPET), have been described in previous publications by the same authors.^47–53^ In this section, the novel six-compartment (6-MPET) formulation (and its FEM-based discretisation) is concisely described. This 6-MPET extension is embedded within a larger workflow and replaces the 4-MPET template developed by the same authors.^3,46–48^ Methodologically, the work presented here builds upon previous work^47–53^ by: (i) defining two additional compartments (reflecting the glial cells and perivascular space/glymphatic system) within the multiple network poroelastic framework in order to more accurately mimic the physiology of the neurovascular unit; and (ii), by partitioning white and grey matter within the cerebral cortex and enforcing a field of weakly varying parenchymal tissue stiffness for the white matter.

In this work, the consolidated workflow is used on one cognitively healthy control subject. This allows one to acquire a biomechanistic understanding into the basic mechanisms of the neurovascular unit (pial/penetrating arterioles, capillaries, ascending venules/veins, see Figure 1) and glymphatic system in the whole parenchyma, in addition to focusing on five important regions of the brain (cortical grey matter (CGM), hippocampus, thalamus, amygdala and brainstem) through key derivative 6-MPET solution fields (perfusion associated with the capillary compartment, tissue swelling and drainage associated with the capillary, CSF/ECF, glymphatic, glial and paravenous spaces).

The newly developed 6-MPET model utilises the following primitive variables in the governing equations (solid matrix/cerebral tissue displacement, **u**, and the pore pressures of six fluid compartments: *p_a_, p_c_, p_e_, p_v_, p_l_, p_g_*):

(1a-g) $$\begin{array}{c} G\nabla^{2}u+\left(G+\lambda \right)\nabla \varepsilon =\alpha_{a}\nabla p_{a}+\alpha_{c}\nabla p_{c}\\ +\alpha_{e}\nabla p_{e}+\alpha_{v}\nabla p_{v}+\alpha_{l}\nabla p_{l}+\alpha_{g}\nabla p_{g}\\ c_{a}{\overset{\cdot }{p}}_{a}+\alpha_{a}\nabla \cdot \overset{\cdot }{u}-\nabla \cdot \left(K_{a}\nabla p_{a}\right)=-Q_{\mathrm{in}}+S_{\mathrm{ca}}+S_{\mathrm{la}}\\ c_{c}{\overset{\cdot }{p}}_{c}+\alpha_{c}\nabla \cdot \overset{\cdot }{u}-\nabla \cdot \left(K_{c}\nabla p_{c}\right)=S_{\mathrm{ac}}+S_{\mathrm{vc}}+S_{\mathrm{lc}}\\ c_{e}{\overset{\cdot }{p}}_{e}+\alpha_{e}\nabla \cdot \overset{\cdot }{u}-\nabla \cdot \left(K_{e}\nabla p_{e}\right)=S_{\mathrm{le}}+S_{\mathrm{ge}}\\ c_{v}{\overset{\cdot }{p}}_{v}+\alpha_{v}\nabla \cdot \overset{\cdot }{u}-\nabla \cdot \left(K_{v}\nabla p_{v}\right)=S_{\mathrm{cv}}+S_{\mathrm{lv}}+Q_{\mathrm{out}}\\ c_{l}{\overset{\cdot }{p}}_{l}+\alpha_{l}\nabla \cdot \overset{\cdot }{u}-\nabla \cdot \left(K_{l}\nabla p_{l}\right)=S_{\mathrm{al}}+S_{\mathrm{cl}}\\ +S_{\mathrm{vl}}+S_{\mathrm{el}}+S_{\mathrm{gl}}\\ c_{g}{\overset{\cdot }{p}}_{g}+\alpha_{g}\nabla \cdot \overset{\cdot }{u}-\nabla \cdot \left(K_{g}\nabla p_{g}\right)=S_{\mathrm{eg}}+S_{\mathrm{lg}} \end{array}$$

**Figure 2.**
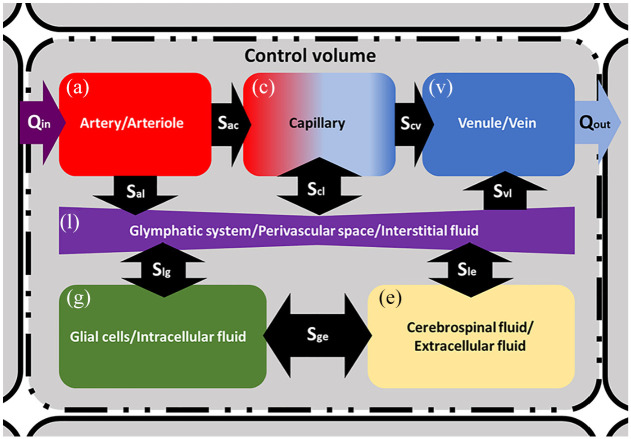
A schematic representation of the 6-MPET model within a control volume. Directional flow constraints are applied between the CSF/ECF (e) and the arterial/arteriole (a), capillary (c) and venule/venous (v) network, as dictated by the physiology of these compartments. The glymphatic system (l) mediates the transfer of fluid between the blood networks associated with the neurovascular unit, the CSF/ECF and the glial cells (g). Directional flow transfer is depicted by the black arrows, and the spatially varying source/sink terms associated with the conservation equations are depicted in the form *S_xy_*.

From equation (1a–g), α is the Biot-Willis coefficient (which satisfies ϕ≤α_*a*_+α_*c*_+α_*e*_+α_*v*_+α_*l*_+α_*g*_≤ 1, where ϕ is the total porosity), *c* the constrained specific storage, ε denotes the dilatational strain, the *S_xy_* terms in equation (1b–g) define spatially varying source (*S_xy_* > 0) or sink (*S_xy_* < 0) densities (rate of fluid transfer between networks driven by a hydrostatic pressure gradient) incorporating the intercompartmental transfer coefficient (ω_yx_, which scales the flow between network *y* and *x*). *Q_in_* and *Q_out_* represent the flux entering, and leaving the control volume, ***K*** is the hydraulic permeability tensor (equal to **κ**(μ^−1^), where **κ** is the permeability tensor and μ is the viscosity) for each of the six fluid compartments. In this work, the CSF/ECF compartment is anisotropic, whilst the remaining fluid compartments are assumed isotropic. For more details, the reader is referred to previous work by the same authors.^47,48,49,54^ Figure 2 depicts the nature of the control volume in which the 6-MPET model operates within. Table 1 lists the parameters used to run the 6-MPET model.

**Table 1.** 6-MPET parameters used in this study.

Parameters	Values	Units	Parameters	Values	Units
α_a,v_	0.1		*c_v,l,g_*	1.5 × 10^−5^	m^2^N^−1^
α_l,g,c,e_	0.2		*c_e_*	3.9 × 10^−4^	m^2^N^−1^
*λ_g_*	505	Pa	*k_a_*_,*e,v*_	1.0 × 10^−10^	m^2^
*G_g_*	216	Pa	*k_l_*	2.0 × 10^−11^	m^2^
*λ_w_*	1010	Pa	*k_g_*	5.0 × 10^−8^	m^2^
*G_w_*	433	Pa	*k_c,white_*	1.0 × 10^−10^	m^2^
*L*	70 × 10^−3^	m	*k_c,grey_*	1.0 × 10^−8^	m^2^
*d*	3 × 10^−3^	m	ω_*ac,cv*_	1.5 × 10^−19^	m^2^N^−1^s^−1^
*p_ls_*	SSBF	Pa	ω_*al*_	2.8 × 10^−14^	m^2^N^−1^s^−1^
*p_lv_*	2445	Pa	ω_*cl,vl,el,gl,eg*_	4.1 × 10^−9^	m^2^N^−1^s^−1^
*p_gv_*	1767	Pa	*R*	8.5 × 10^13^	m^−3^
*p_bp_*	650	Pa	*Q_p_*	5.8 × 10^−9^	m^3^s^−1^
*c_a_*_,*c*_	2.9 × 10^−4^	m^2^N^−1^	Δt	0.1	s

Values of shear modulus, *G*, and Lamé’s constant λ for the white matter (with subscript *w*) than the grey matter (with subscript *g*) are given. The reader is referred to Vardakis et al., Guo et al. and Chou^3,47,48,54–56^ for further details.

SSBF: subject-specific blood flow profile derived arterial pressure.

### The 6-MPET numerical template

The 6-MPET system of partial differential equations has been discretised using the finite element method (FEM). The FEM code was written in FORTRAN 90/95. In previous work, the authors conducted grid-independence studies, verified the multiporoelastic system against analytical solutions,^47,57,58^ in addition to the system being recently validated with respect to data arising from both a CSF infusion test^54^ and ASL based values.^55^

In line with previous work, grid-independence of the 6-MPET system was conducted, and converged solutions fell within a specified percentage band of numerical error estimates. For details regarding the discretisation of the conservation equations, the reader is referred to Guo et al. and Vardakis et al.^47,48,54,55^ Finally, the discretised form of the 6-MPET system is solved using the PETSc library.^59,60^

### Subject-specific dataset, 6-MPET parameters and boundary conditions

#### Subject-specific dataset

The details regarding the subject-specific dataset used in the execution of the 6-MPET model presented here, along with further details can be found in previous publications by the same authors.^3,47,48^ Subject and region-specific geometries of the cortical tissue and cerebroventricular system, permeability tensor maps of the cerebral tissue and subject-specific flow waveforms were acquired using the protocol in Vardakis et al. and Guo et al.^3,47,48^ From this combined dataset, a 72-year-old cognitively healthy female subject (whilst undergoing a period of high activity) was used to execute the high-precision workflow.

#### Parameters of the 6-MPET system

As in previous work,^55^ the differentiation between grey and white matter is adopted, as the segmentation of the of T1w MR images defines 28 separate regions in both hemispheres, including the grey and white matter. This partitioning of the parenchymal volume allows for different values to be assigned for the mechanical properties (such as Young’s modulus) of the grey (584 Pa)^47–51^ and white matter (1168 Pa).^55^ In order to further extend the state-of-the-art, this numerical template also allows for the perturbation of the Young’s modulus field within the white matter, of the order of ±10 Pa. It has been observed that a decrease in brain viscoelastic parameters is associated with ageing,^61^ and in AD, the overall brain stiffness is observed to decrease.^62,63^ To be able to account for these key characteristics within the presented numerical template, the 6-MPET code was augmented with the capability of capturing a spatially varying elasticity field in the white matter. Additionally, the permeability of the arteriole/capillary compartment is assigned partitioned values for white (*k*_c,white_) and grey matter (*k*_c,grey_), and reflects the calibration of the capillary compartment with respect to the validation of the filtration velocity via CBF data acquired from ASL images.^55^ In this manuscript, a new set of Biot-Willis constants were used, reflecting the theoretical nature of this study. In previous studies (see Vardakis et al.^48^ for example), the value of the CSF/ISF compartment in the 4-MPET framework was almost double (0.49) that of the feeding blood compartments (arterial and capillary compartments were set at 0.25), and approached small values for the less compliant venous system (10^−2^). In this work, the larger calibre vessels (arteries/arterioles and venules/veins) that feed and drain the representative elementary volume of the 6-MPET system are scaled to be lower than the constituents of the NVU (see Table 1), in order to simplify the study of the 6-MPET system, and also to reflect the need to physiologically define the low resistance pathways facilitating CSF flow (paravascular spaces surrounding leptomeningeal arteries).^64^ The intercompartmental transfer coefficients (ω_al_, ω_cl_, ω_vl_. ω_el_, ω_gl_, ω_eg_) associated with the two additional compartments (glial cells/intracellular fuid and glymphatic system/perivascular space/ISF) are calculated by incorporating parameters associated with hydraulic conductivity, a bidirectional constrained specific storage coefficient and the surface to volume ratio. These terms also possess the ability to incorporate aquaporin expression (see Chou^56^ for further details). The remaining intercompartmental flux terms (ω_ac_, ω_cv_) are the same as in previous studies (see for instance Vardakis et al. and Guo et al.).^3,47,48,54,55^

#### Boundary conditions of the 6-MPET numerical template

In the work conducted here, the outer/inner boundary of the cerebral tissue represents the cortical surface/cerebroventricular wall respectively. Table 2 lists the Dirichlet, Neumann and Robin boundary conditions used in this model. The details and physiological significance of the boundary conditions associated with the 4-MPET model are explained in previous publications.^3,47–50,52,55^ In this section, only the boundary conditions associated with the two additional compartments (glial and glymphatic system) are described in further detail. In the 6-MPET compartment associated with the glymphatic system, it is assumed that a peak arterial pressure on the cortical surface prevails (∼100 mmHg, in line with previous studies),^47,48,55^ whilst on the surface of the cerebral ventricles, the pressure is equal to that of the average pressure associated with the capillary compartment (∼18 mmHg).^55^ This is not an unreasonable assumption to make, since it is assumed that the perivascular space maintains its pressure throughout the parenchymal volume in order to maintain cerebral homeostasis and the integrity of the BBB. For the glial cell compartment, it is assumed that a Dirichlet condition equal to the average of the CSF/ECF and capillary pore pressure exists on the surface of the cerebral ventricles, whilst the skull has the CSF/ECF pore pressure as a boundary condition. These assumptions are made on the premise that within the parenchymal tissue, a continuous CSF compartment exists, spanning from the SAS, cisterns, and paravascular spaces, whilst simultaneously considering the parenchymal vasculature.^41^

**Table 2.** Boundary conditions used in the 6-MPET model. The reader is referred to Vardakis et al., Guo et al. and Chou^3,47,48,54–56^ for further details. ∂Γs and ∂Γv are boundaries at the skull and cerebroventricular wall respectively, **n** is the outward unit normal vector and *u*_1_ is the maximum ventricular displacement at each time increment.

6-MPET variable	Cortical surface	Cerebroventricular wall
u	**u** = **0**	$-p_{V}n=\sigma_{\mathrm{ij}}\cdot n$
*p_a_*	$\nabla p_{a}n=Q_{a}$	$\nabla p_{a}n=0$
*p_c_*	$\nabla p_{c}n=0$	$\kappa_{c\to \mathrm{vent}}\nabla p_{c}n=-Q_{p}$
*p_e_*	$p_{e}=p^{v}+\mu^{e}RQ_{o}$	$Q_{p}=\frac{\pi d^{4}}{128\mu_{e}L}\left(p_{e}{|}_{\partial \Gamma_{V}}-p_{e}{|}_{\partial \Gamma_{S}}\right)-4\pi k_{e}\left(r_{v}+u_{1}^{n}\right)\nabla p_{e}n+4\pi {\left(r_{v}+u_{1}^{n}\right)}^{2}\overset{\cdot }{u}$
*p_v_*	$p_{v}n=p_{\mathrm{bp}}$	$\nabla p_{v}n=0$
*p_l_*	$p_{l}=p_{\mathrm{ls}}$	$p_{l\prime }=p_{\mathrm{lv}}$
*p_g_*	$p_{g}=p_{e}$	$p_{g\prime }=p_{\mathrm{gv}}$

The reader is referred to previous work^3,46–48^ regarding the methodology behind the implementation of the boundary conditions, including the Neumann application of the CBF waveforms associated with the driving compartment (the arterial compartment) of the 4-MPET (and by extension, the 6-MPET) system. The solutions of the 6-MPET system are acquired from the final periodic steady state (spanning 50 simulation cycles). It should be noted that for this subject, the internal carotid artery (ICA) profiles between left and right hemisphere were closely aligned (unlike the variation present for other subjects studied in work by the same authors).^47,48^ The VA profiles are identical for both hemispheres, in line with previous work.^47,48^ This implies a greater degree of morphological symmetry to the developing solution fields (as the arterial compartment is the driving compartment of the system) and allows the compartmental interrogation of the 6-MPET model to be conducted according to the aforementioned extensions to the high precision workflow.

## Results and discussion

**Figure 3.**
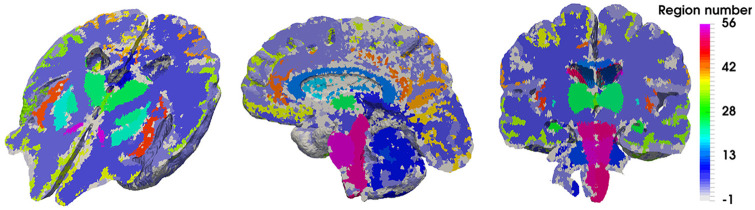
Representation of the regional parenchymal specificity incorporated in the high precision workflow. A sagittal, coronal and perspective representation of the axial plane is shown in this figure, highlighting the 56 regions (28 for each hemisphere) of the parenchyma that can assist in stratifying the 6-MPET results.

Figure 3 depicts the 28 regions (spanning the two hemispheres, so a total of 56 separate regions) that this precision medicine pipeline can stratify results for (like the hippocampus, brainstem and thalamus), in addition to the outputs of the 6-MPET system for the chosen subject (72-year-old female cognitively healthy control subject).

**Figure 4.**
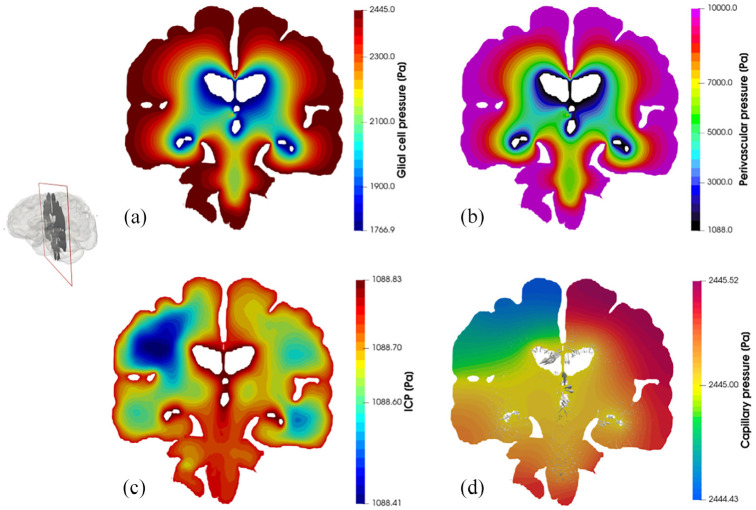
Coronal slices of the 6-MPET solution fields allied to the parenchymal volume of the 72-year-old cognitively healthy female subject undergoing a period of high activity. Both the pore pressure of the glial cell compartment (a) and the perivascular/glymphatic system (b) is symmetric in morphology, reflecting the nature of the boundary conditions imposed for these compartments whilst also considering the intercompartmental influences of the remaining compartments. The latter influences are more prevalent in the compartments describing the (c) intracranial pressure (CSF/ECF compartment) and (d) capillary pressure, since the former superimposes a highly distinctive permeability tensor map which can capture the underlying parenchymal microstructure (see Guo et al. and Vardakis et al.^47,48^ for more details). The capillary compartment and its accompanying pore pressure reflect the asymmetric nature associated with the arterial flow variability being applied on the partitioned parenchymal surface, as it is in direct communication to the high transmantle pressure gradient present in that compartment. The superimposed velocity vectors associated to the paravascular/glymphatic system filtration velocity assist in attenuating the level of detail associated with this rich dataset.

**Figure 5.**
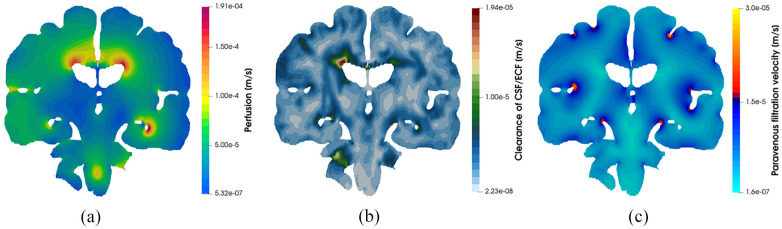
Coronal slices of the 6-MPET solution fields allied to (a) perfusion, (b) clearance of CSF/ECF and (c) paravenous filtration velocity. Some quantitative similarities for these solution fields can be found in Vardakis et al., Croci et al. and Thomas.^48,65,66^ It can be seen that the impact of the cerebroventricular geometry (in terms of its concavity) influences the localisation of the areas of peak perfusion, paravenous filtration, and clearance of CSF/ECF. These areas could play a role in AD progression.

Figure 4 depicts core pore pressure solution fields garnered from executing this workflow, including glial and perivascular pressure (Figure 4(a) and (b)), intracranial pressure (ICP) (Figure 4(c)), and capillary pressure within the parenchymal volume (Figure 4(d)). Figure 5 depicts additional derivative solution fields (obtained at the post-processing stage of the workflow)^47,48,55^ of interest, namely perfusion (Darcy velocity of the capillary compartment, Figure 5(a), clearance of CSF/ECF (Darcy velocity of the CSF/ECF compartment, Figure 5(b) and paravenous filtration velocity (Darcy velocity of the venule/venous compartment, Figure 5(c). Clearance of CSF/ECF emphasizes the intertwined nature of the 6-MPET compartments, as it incorporates the effects of the subject-specific permeability tensor maps.

**Figure 6.**
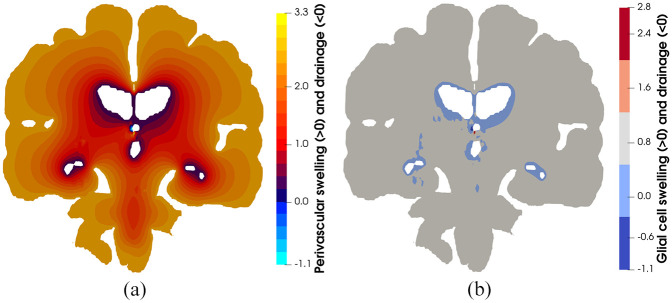
(a) Perivascular and (b) glial cell swelling and drainage. Swelling in depicted from positive values of the respective compartmental fluid content, whilst drainage is depicted with negative values. It is interesting to note the influence of the cerebroventricular system (as it possesses a high concavity) in accumulating strain, which is reflected in the highly accurate qualitative depiction of periventricular lucency, a potential inhibitor to the transport pathways associated with the glymphatic system (perivascular and glial cell swelling). On the contrary, drainage in these solution fields may be representative ependymal cell breakdown (which is assumed to be fused with the surrounding glial cells), forcing fluid to leave the ventricles in order to relieve the underlying pressure burden in these cavities.

Figures 4 and 5 highlight the asymmetric nature of the primary solution fields allied to ICP, and capillary pressure, in addition to the derivative fields associated with fluid content and Darcy filtration velocity for the capillary, CSF/ECF, venous, and glial cell compartments. Derivative solution fields can be seen to capture the inherent concavity of the cerebral cortex and cerebroventricular system (especially in the periventricular regions), and in doing so, highlight the ability of the numerical template to incorporate areas of accumulating strain and scaled pore pressure fields with high qualitative accuracy. Periventricular lucency (PVL)^67^ (accompanied by localized cerebroventricular rupture)^68^ is a common neuroradiological finding, yet its pathogenesis and clinical significance remains inconclusive.^69^ It is postulated that the relief of excess pressure within the cerebroventricular system is facilitated by the tactical breakdown of the ependymal surface which is thought to also involve AQP4 (see Figure 1d).^70^ Additionally, the concavity of the cerebroventricular system^47,48,52,69,71^ is a constituent of PVL, and may also involve CSF/ECF clearance (see Figure 5) which may utilise stretch/swelling-activated Cl^−^ channels (and therefore the extent to which the microglia can monitor the cerebral parenchyma).^72^ In this work, PVL is calculated as the increased fluid content in the periventricular regions and can be seen to reflect the aforementioned hallmark characteristics (see Figure 6, perivascular and glial cell swelling and drainage solution fields). Both the anterior and posterior horns of the lateral ventricles portray the focal areas of peak PVL, and these areas coincide with the cerebroventricular regions of pronounced concavity. Previous work by the same authors fortify these qualitative findings.^47,48,50,52^

Table 3 provides a snapshot of the additional capabilities of this consolidated pipeline. Derivative quantities of interest (perfusion, and degree of swelling and drainage for the perivenous, glial, glymphatic, CSF/ECF and capillary compartment) are given for five important regions of the brain (cortical grey matter,^3^ hippocampus,^48^ thalamus, amygdala and brainstem) that are important in the trajectory of AD progression. Developing protein pathology in the medial temporal lobe triggers cognitive deficit in AD, hence the focus on hippocampal^73^ dysfunction and how memory symptomology^74^ can be alleviated by therapies targeting this region. In this work, the thalamus is analysed for its perfusion and fluid content variability over key compartments of the 6-MPET system, since interrogating this region is deemed to provide a more balanced understanding of the transition between MCI and AD.^74^ Regional thalamic degeneration in AD is emerging as an important area of consideration alongside the hippocampus and amygdala.^75^ There is MRI evidence linking internal medullary lamina atrophy which is thought to promote thalamic degeneration in AD patients.^75^ The results in Table 3 show that although this region neighbours the hippocampus, its location and geometrical structure promotes peak perfusion values that vary (∼62 µm/s compared to ∼84 µm/s in the hippocampus) and is accompanied by a high proportion of paravenous drainage and swelling, whilst solely portraying swelling characteristics for the glial and capillary compartments. Like the thalamus, the amygdala is also affected in the nascent stages of AD, and evidence exists linking a reduced intrinsic functional connectivity in this region.^76^ It was observed that this region portrayed swelling characteristics in the capillary and glial compartments, and also maintained a large perfusion range (∼25 µm/s–0.2 mm/s), which could prove to be an interesting biomechanistic biomarker considering imaging studies have consistently shown evidence of atrophy in this region.^75^ The consistent swelling in the NVU in all regions considered may also signify an increased likelihood of BBB breakdown/dysregulation, which may ultimately lead to enhancing the malignantly progressive burden of neurodegeneration. This can include the perivascular accumulation of neurotoxic products and the promotion of small vessel disease–related brain tissue injury.^77^

**Table 3.** 6-MPET results for derivative quantities for five different regions of the brain.

Region	Perfusion (min, max)	Fluid swelling (>0) & drainage (<0) for different compartments				
		Capillary	CSF/ECF	Glymphatic	Glial	Paravenous	
CGM	4.3 µm/s, 0.7 mm/s	[−0.3,1.1]	[−0.7,0.8]	[−0.7,3.0]	[−0.5,1.1]	[−0.4,0.3]	
Hippocampus	0 m/s, 0.19 mm/s	[−0.6,1.1]	[−0.9,0.8]	[−0.9,2.9]	[−0.7,1.0]	[−0.7,0.3]	
Thalamus	0 m/s, 62.2 µm/s	[0.2.1.1]	[−0.2,0.8]	[−0.04,2.7]	[0.1,0.9]	[−0.7,0.3]	
Amygdala	25.6 µm/s, 0.2 mm/s	[0.2,0.8]	[−0.2,0.5]	[−0.2,2.6]	[0.005,0.8]	[−0.2,0.3]	
Brainstem	5.3 µm/s, 0.2 mm/s	[0.2,1.0]	[−0.1,0.7]	[−0.1,2.9]	[0.03,1.0]	[−0.1,0.3]	

CGM: cortical grey matter.

Brainstem nuclei regulate autonomic, cognitive, and behavioural functions. Therefore, this region may be playing an important role in AD progression.^78^ Throughout the developmental stages of AD, brainstem neurodegeneration has been linked to a disruption in sleep patterns,^79^ respiration and blood pressure.^80^ Additionally, it is known that the proportion of 3R tau in the brainstem neurofibrillary changes increases with disease progression.^81^ Considering its location (its close proximity to the cerebellum), it is unsurprising that this region occupies a broad perfusion range (∼5 µm/s–0.2 mm/s), as the partitioned arterial boundary conditions (in Neumann form) on the cortical surface of the parenchyma approach 100 mmHg,^3,47,48^ reflecting the driving flow rates of over 700 ml/min for the left and right ICA, and approaching 200 ml/min for the left and right VA during peak systole within the fifty simulated cycles. Interestingly, the brainstem portrays evidence of paravenous drainage, but at the expense of predominant swelling in the paravascular space, glial cells, CSF/ECF and the capillary compartments.

The cortical grey matter (CGM) displays similar characteristics, with the notable exception of additional measurable drainage in the CSF/ECF and a larger peak perfusion velocity of 0.7 mm/s, as opposed to 0.2 mm/s in the brainstem. The blood perfusion results obtained from the 6-MPET model presented here lie within a similar quantitative range as the perfusion results obtained from a simpler multiporoelastic model that adhered to ranges that mimicked arterial spin labelling (ASL)-derived perfusion.^55^ Focusing on the CGM in this study, one is able to gain theoretical insight into the glymphatic system in this region, and its interplay with the other constituent compartment that define the representative elementary volume of parenchymal tissue. The enlarged perivascular space during AD postulated by a recent study^82^ can be given further consideration, since this work relaxes the assumption that the enlarged perivascular space occurs as a direct result of the periarterial swelling,^3^ and instead allows a similar statistical analysis to be conducted on the compartments that reflect these intertwined alterations most accurately (glymphatic and capillary compartment).

### Limitations of the study

In this extensive, yet preliminary study, only one subject from the Lido study cohort was analysed (execution of the pipeline for the remaining cases is currently in progress). The emphasis has been placed on being able to associate some of the resulting outputs of the consolidated framework outlined in §2, with a potential setup to acquire AD specific biomarkers that respect the theoretical underpinning of the neurovascular unit in its entirety. Importantly, the work presented here will form part of a broader study, which will involve the assessment of the consolidated pipeline utilising the entire Lido dataset.^3,47,48^

Important limitations include the omission of cross-storage effects from the MPET formulation.^49,83^ The components of cerebroventricular displacement were constrained (rigid skull boundary condition) on the cortical surface (thereby omitting the cortical SAS), and the 6-MPET field equations are formulated by using a linear-elastic constitutive law (which is not unreasonable for chronic neurodegenerative diseases, but it is a limitation nonetheless). Regarding the additional compartments of the poroelastic model, the lack of additional experimental data to validate the full spectrum of model parameters required for all six compartments of the poroelastic model is a limitation of this study. Additionally, the numerical template omits body forces and inertial terms in the conservation equations since the acceleration frequencies are low in biological flows.^49,51^ Further limitations are discussed in Guo et al., Vardakis et al., Tully and Ventikos and Chou et al.^47–53^

## Conclusion

This work outlines a novel 3D six-compartment poroelastic system for modelling the neurovascular unit by incorporating glial cells and the newly discovered glymphatic pathway. Additionally, this model is embedded within a high precision workflow^3^ that has been extended by allowing for the variation in mechanical properties (parenchymal tissue stiffness) in important regions of the brain (white matter). This paves the way for detailed studies allied to ageing induced neurodegeneration (such as AD) and allows for the probing and exploration of the highly dynamic and multifactorial fluid transport system that exists within the brain. This workflow is used on one cognitively healthy control subject in order to explore the underlying mechanisms of the neurovascular unit in five important regions of the brain (cortical grey matter, hippocampus, thalamus, amygdala and brainstem) through the regional differences in derivative solution fields of the multiporoelastic model (such as perfusion, swelling and drainage of the neurovascular unit).

Interesting and useful extensions to this numerical template include the coupling of this high precision workflow with a robust fluid flow solver that will help capture the ageing, lifestyle and neurodegenerative influences on cerebroventricular flow complexity, and incorporate novel boundary conditions on the inner surface of the cerebroventricular wall that capture cilia-driven flow within these important cavities. Finally, incorporating the Donnan effect within the 6-MPET numerical formulation (see Vardakis et al.^52^ for references therein) is also considered an important extension to the current numerical template.
